# Global Surgery – Informing National Strategies for Scaling Up Surgery in Sub-Saharan Africa

**DOI:** 10.15171/ijhpm.2018.27

**Published:** 2018-04-08

**Authors:** Jakub Gajewski, Leon Bijlmakers, Ruairí Brugha

**Affiliations:** ^1^Royal College of Surgeons in Ireland, Dublin 2, Ireland.; ^2^Radboud University Medical Centre, Nijmegen, The Netherlands.

**Keywords:** Global Surgery, Africa, Systems Approach, National Surgical Plans

## Abstract

Surgery has the potential to address one of the largest, neglected burdens of disease in low- and middle-income countries (LMICs), especially in sub-Saharan Africa (SSA). The Lancet Commission on Global Surgery (LCoGS) has provided a blueprint for a systems approach to making safe emergency and elective surgery accessible and affordable and has started to enable African governments to develop national surgical plans. This editorial outlines an important gap, which is the need for surgical systems research, especially at district hospitals which are the first point of surgical care for rural communities, to inform the implementation of country plans. Using the Lancet Commission as a starting point and illustrated by two European Union (EU) funded research projects, we point to the need for implementation research to develop and evaluate contextualised strategies. As illustrated by the case study of Zambia, coordination by global and external stakeholders can enable governments to lead national scale-up of essential surgery, supported by national partners including surgical specialist associations.

## Global Surgery - the Current Situation

It is approaching three years since the Lancet Commission on Global Surgery (LCoGS) published *Global Surgery: evidence and solutions for achieving health, welfare and economic development.*^1-4^ This is an opportune point to take stock of the direction and progress in scaling up essential surgical care in sub-Saharan Africa (SSA), the region with the greatest need and lowest response capacity.^5^ The case for investing in making safe surgery accessible to underserved communities is compelling: 5 billion people lack access to safe, affordable surgical and anaesthetic care, with low- and middle-income countries (LMICs) worst affected^6^; and 33 million people endure catastrophic expenditure each year in paying for essential, often life-saving surgery.^1^ There is evidence that essential surgery can be delivered safely, cost effectively and is affordable and feasible, even in low resource settings.^1,7,8^ However, in much of SSA, elective and emergency general surgery is only available in the few urban hospitals that are staffed by specialist surgeons.^1,9^ District level hospitals (DLHs) in Malawi and Zambia and many other African countries deliver emergency obstetrical interventions – mainly caesarean sections, and occasional hysterectomies, as well as limited general surgery – mainly hernia repairs, but also laparotomies and hydrocoele repairs.^10^ In Zambia, such surgery is often undertaken by non-physician clinicians (NPCs) and medical officers with limited surgical skills and experience, lacking support and supervision and working in an uncertain legal and regulatory environment.^11^ Yet for rural populations they are often the only accessible source of elective and life-saving emergency surgery.^11^

The LCoGS produced a blueprint, outlining the inputs, processes and systems needed for the provision of safe surgical and anaesthesia care, including^1^: trained staff; essential infrastructure and equipment; reliable supply chains, sterilisation and blood supplies; information systems and financing mechanisms; and referral and care delivery protocols to minimise delays for communities and patients in receiving appropriate care. Because of the limited research evidence, the Commission relied on expert opinion and modelling of scarce existing data, framed by a comprehensive health systems analysis.^1,12^ It proposed three ‘must do,’ ‘bellwether procedures’ that first-level hospitals should be able to undertake – notably caesarean sections, laparotomies and management of open fractures; and a longer list of ‘should do’ and ‘can do’ procedures, to be undertaken at first level or referral hospitals, depending on the context.^1^ This paper looks critically at the link between two dimensions of global surgery, where the LCoGS made recommendations and where there has been some, albeit slow, progress in SSA in the last 2-3 years: the development of national surgical plans and the research agenda for informing the development, implementation and sustainable scale-up of surgical systems.

## Progress Made - National Surgical Plans

Up to 2015, the target year for reaching the millennium development goals and the year when the LCoGS published its report,^1^ only 2% of 4064 health targets in national health strategic plans from 43 African countries covered surgical conditions or care; and 33% of national health policies had no surgical targets.^13^ The LCoGS proposed five dimensions as a framework for the development of National Surgery, Obstetric and Anaesthesia Plans: infrastructure, workforce, service delivery, information management and financing.^1^ The Harvard Program in Global Surgery and Social Change (PGSSC) has played a key role in the development of the national surgical plan in Zambia in 2015-2016.^14^ The plan was developed through reviews of national level data and semi-structured interviews of country specialists across the disciplines of surgery, obstetrics, and anaesthesia; and representatives of relevant government ministries; and national and international partners. The Harvard Program is also supporting the development of national plans in Ethiopia, Rwanda, Nigeria, Tanzania,^15^ and in Madagascar.^16,17^

## Implementation Priorities and Risks

While country national surgical plan design processes have been consultative, and rightly so, a lack of contextualised research findings to inform them has been a weakness and it remains a gap in respect to plans for evaluating implementation.^14^ Without a strong empirical base, there is a risk that national surgical plans will be unrealistic or remain aspirational.^18^ Surgical plans need a clear implementation strategy that involves local champions, under the leadership of governments, with strategic links to global leaders and funding agencies. Implementation strategies should be based on an understanding of national surgical systems as complex adaptive systems^19^; and should incorporate a surgical systems research arm for testing the feasibility of solutions in large population sites. Some countries, such as South Africa, have the capacity to bring together all these components,^20^ with a contextualised implementation and research agenda.^21^ Others may benefit from global-local research partnerships where large scale or complex research, as well as associated resource mobilisation, is required.^22^ Lessons from implementing the Millennium Development Goals (MDGs) and other global indicator strategies can inform investment choices and implementation priorities, ensuring that progress is measured and that governments (and global stakeholders) are held accountable by citizens.^23,24^

Given the scarcity of empirical research on the reality and potential for making surgery available to rural populations through DLHs, the risk is that the voice of district stakeholders may not be represented in the consultations that inform national surgical plans.^25^ Rural and district areas are the setting for most unmet population surgical need^1,26,27^; and new research evidence is emerging that makes the case for investing in district level surgical care.^10,11,28,29^ This can reduce the demands on referral hospitals, allowing specialist surgeons to deliver more complex surgery^26^; and enable DLHs to deliver the ‘must do’ and potentially many of the ‘should do’ procedures.^30,31^ In countries where surgery is undertaken by non-specialists, training and quality assurance systems are essential. Clear career paths are also needed to ensure professional progression paths exist for surgically trained NPCs and medical officers at the district level, reducing the need to pursue careers in urban areas.^11^ In many African countries, specialist surgeons lack the resources to undertake supervisory visits and have little direct contact with or experience of DLHs, which may lead them to underestimate the current scale and potential for district surgery^10^; and, as a result, to recommend that resources be channeled first to referral hospitals. The dearth of empirical research on surgical capacity at DLHs^11^ has meant that the debate on how rural populations can be served and who should deliver essential surgery to them – specialist surgeons, general medical officers or surgically trained NPCs – has become politicised.^32,33^

## Research for Change

The LCoGS identified some of the rate limiting steps and called for a systems research agenda to underpin the development and implementation of national surgical plans, emphasising the need to identify and test contextualised responses.^1^ Yet, despite a World Health Assembly resolution in 2015, calling for surgical systems scale-up globally,^34^ there is still limited global policy attention to surgery in early 2018. This gap means there is a window of opportunity to coordinate research initiatives in support of nationally-led surgical systems development so as to: (*i*) inform national surgical plans, (*ii*) test innovative solutions; and (*iii*) provide feedback to inform national scale-up, based on evaluations of feasibility, quality and safety, affordability, cost-effectiveness and health impact. Subramanian and colleagues’ systematic review evaluated conceptual participatory action research models that involve country stakeholders in learning by doing, incorporating the critical factors that determine how the results of pilot projects feed into national programmes.^35^ The authors identified and evaluated six models for scaling-up health services and concluded that there is no ‘one size fits all’ optimal approach as strategies need to consider the political, organisational and functional dimensions of scale-up, supporting national stakeholders and developing local organisational capacity.^35^ Successful models place less emphasis on initial planning, and more on facilitating implementation and on “learning by doing, embracing error, and linking knowledge-building with action as implementation is occurring.”^35^

Drawing on this review,^35^ we propose the building of country networks under the coordination of national ministries of health, supported by regional and global surgical and systems research stakeholders. The critical processes, when applied to surgical systems scale-up, should comprise: (*i*) leadership from ministries of health and national surgical specialist bodies, supported by surgical systems researchers; (*ii*) identification of workforce, other capacity and resource obstacles; and enablers to the delivery of essential surgical services through networks of district and referral hospitals; (*iii*) collaborations between ministries, researchers, non-governmental organisations and specialist surgeons in implementing and evaluating training and supervision interventions, involving government approved surgical clinicians at DLHs – be they NPCs or doctors; (*iv*) feedback loops to allow continuous adaptation of interventions to local contexts, informing the development and adjusting the implementation of national surgical plans; and (*v*) the use of implementation (mixed methods) research, combining rigorous quantitative measurements of cost, surgical outputs and outcomes, with qualitative explanatory evaluations of the complex interventions and processes needed to quality assure and scale up surgical services. In addition, as outlined by the Commission,^1^ countries need to invest in surgical infrastructure, purchase and maintain essential equipment, develop reliable supply chains, and ensure that financial mechanisms enable citizens to access surgical care regardless of social status.

## Case Study of Zambia

Zambia provides an interesting case where two independent processes for scaling up a national surgical service, both under the guidance of Government, developed between 2011 and 2016. Here we draw on our experience of developing, implementing and evaluating a national surgical training and supervision intervention in Malawi and Zambia, as part of the Clinical Officer Surgical Training project (COST-Africa), 2011-2016. Lessons were used to design a new implementation research project to scale up safe surgery for district and rural populations (SURG-Africa) in Malawi, Zambia, and Tanzania, 2017-2020. The European Union (EU) awarded 9 million euros to support these research projects.

The COST-Africa project, which was originally conceived and funded as research of a national NPC training intervention, was adapted in Zambia in 2012, as advised by national ministries, to focus on supervision of existing surgically trained NPCs. Ten NPC graduates were deployed during 2013-2014 as surgical clinicians in nine DLHs across nine different provinces, supervised by four provincial specialist surgeons. In 2015, representatives of the local implementing partner, the national Surgical Society of Zambia, were invited to join a national working group that was tasked with drafting Zambia’s national surgical plan. Thereby, lessons from COST-Africa, specifically a well-documented supervision model, were incorporated into the national surgical plan in 2016, which was launched in 2017.^14^ One of the erstwhile provincial surgeons, who had road-tested the COST-Africa supervision model, was appointed Deputy National Director for Clinical Services in 2017, tasked with implementation of the national plan. At a meeting in March 2017 of the Ministry of Health with the new SURG-Africa project team, again led locally by the Surgical Society of Zambia, it was agreed that the new SURG-Africa project would conduct an in depth-evaluation of the new supervision model, comprising remote and in-the-field supervision of surgical services at DLHs.

As Paina and Peters state^19^: “the processes or pathways for introducing and scaling up interventions can be as important as the content of the intervention itself… (and need to comprise) highly heterogeneous groups of actors.” In Zambia, there has been a serendipitous confluence of events, including: (*i*) responsibility for district hospitals was restored to the Ministry of Health in 2015 after a gap of three years, during which responsibility lay with the Ministry of Community Development & Maternal and Child Health; (*ii*) a collaborative approach by the Harvard Global Program and the Zambia Ministry of Health, including surgical champions, led to the development of a national surgical plan; and (*iii*) implementation of the two large EU-funded surgical systems research projects, one generating lessons to inform the plan and the second providing an opportunity to evaluate implementation. While there is debate on the sequencing of investments in national and referral hospitals vis-à-vis district level investments, the processes of developing national surgical plan as a blueprint for scale up of surgical services have been collaborative and have avoided some negative features of global health initiatives that impose disease specific interventions on countries with little regard to context or sustainability.^19,36-38^ However, if developed without an adequate evidence-base and if implemented without in-built feedback loops,^19^ implementation failure or unsustainable programmes are real risks.

In Zambia, where the national surgical plan is due to be implemented, and in Tanzania, where such a plan is being developed in 2017-2018, SURG-Africa aims to test the components of a scalable national surgical system in 2017-2020, feeding emerging results to national ministries and their partners, from early 2018. The COST-Africa and SURG-Africa coordinator at the Royal College of Surgeons in Ireland and the coordinator of the Harvard Program in Global Surgery have established links, enabling coordinated support to country plans by ‘northern’ partners. However, little can be achieved without the engagement of country stakeholders, through national surgical associations working with and under the leadership of Ministries of Health. Participatory action research focusing on implementation of national surgical plans can then provide the mechanism for translating evidence on the feasibility, cost-effectiveness, impact and potential for scale-up of tested strategies into sustainable national programmes for making safe surgery accessible to district and rural populations in Africa.

## Ethical issues

Not applicable.

## Competing interests

Authors declare that they have no competing interests.

## Authors’ contributions

All authors contributed substantively to all drafts. All authors read and approved the final manuscript. RB conceived the concept of the paper and drafted the first sketch. KG led on the development of the manuscript with inputs from LB and RB.

## Authors’ affiliations

^1^Royal College of Surgeons in Ireland, Dublin 2, Ireland. ^2^Radboud University Medical Centre, Nijmegen, The Netherlands.
